# Multi-locus DNA sequence analysis, antifungal agent susceptibility, and fungal keratitis outcome in horses from Southeastern United States

**DOI:** 10.1371/journal.pone.0214214

**Published:** 2019-03-28

**Authors:** Megan Cullen, Megan E. Jacob, Vicki Cornish, Ian Q. VanderSchel, Henry Van T. Cotter, Marc A. Cubeta, Ignazio Carbone, Brian C. Gilger

**Affiliations:** 1 Department of Clinical Sciences, NC State University, Raleigh, NC, United States of America; 2 Department of Population Health and Pathobiology, NC State University, Raleigh, NC, United States of America; 3 Center for Integrated Fungal Research, College of Agriculture and Life Sciences, NC State University, Raleigh, NC, United States of America; Leibniz-Institut fur Naturstoff-Forschung und Infektionsbiologie eV Hans-Knoll-Institut, GERMANY

## Abstract

Morphological characterization and multi-locus DNA sequence analysis of fungal isolates obtained from 32 clinical cases of equine fungal keratitis (FK) was performed to identify species and determine associations with antifungal susceptibility, response to therapy and clinical outcome. Two species of *Aspergillus* (*A*. *flavus* and *A*. *fumigatus*) and three species of *Fusarium* (*F*. *falciforme*, *F*. *keratoplasticum*, and *F*. *proliferatum*) were the most common fungi isolated and identified from FK horses. Most (91%) equine FK *Fusarium* nested within the *Fusarium solani* species complex (FSSC) with nine genetically diverse strains/lineages, while 83% of equine FK *Aspergillus* nested within the *A*. *flavus* clade with three genetically diverse lineages. Fungal species and evolutionary lineage were not associated with clinical outcome. However, species of equine FK *Fusarium* were more likely (*p* = 0.045) to be associated with stromal keratitis. Species of *Aspergillus* were more susceptible to voriconazole and terbinafine than species of *Fusarium*, while species of *Fusarium* were more susceptible to thiabendazole than species of *Aspergillus*. At the species level, *A*. *fumigatus* and *A*. *flavus* were more susceptible to voriconazole and terbinafine than *F*. *falciforme*. Natamycin susceptibility was higher for *F*. *falciforme* and *A*. *fumigatus* compared to *A*. *flavus*. Furthermore, *F*. *falciforme* was more susceptible to thiabendazole than *A*. *flavus* and *A*. *fumigatus*. These observed associations of antifungal sensitivity to natamycin, terbinafine, and thiabendazole demonstrate the importance of fungal identification to the species rather than genus level. The results of this study suggest that treatment of equine FK with antifungal agents requires accurate fungal species identification.

## Introduction

Fungal keratitis (FK) is a severe, progressive, inflammatory ocular disease resulting from invasive growth of fungi into the cornea. Fungal keratitis is challenging to manage and can lead to blindness or loss of the affected eye.[1] The incidence of human FK has increased in the past several decades.[2, 3] In subtropical areas, fungal infections are reported to cause up to 35% of all documented keratitis cases in humans, especially in China and India.[1, 3, 4] Fungal keratitis is less common in the US where it is predominantly observed in south Florida and Texas.[2, 5] Nearly half of the causative organisms in FK are filamentous fungi, predominantly species of *Aspergillus* and *Fusarium*, of approximately equal frequency, followed in incidence by species of *Candida*, a dimorphic yeast.[1, 3–5] *Fusarium* spp. and *Aspergillus* spp. accounted for 31% and 25% of filamentous FK isolates from South India; [4] 28% *Fusarium* spp. and 22% *Aspergillus* spp. from East India; [3] and 48% *Fusarium* spp. and 19% *Aspergillus* spp. from Northeast China. [1] In these studies, the fungal species associated with FK were not identified.

Filamentous fungi and yeasts are part of the normal ocular surface microbiome, are soil saprobes and plant pathogens, and thought to be opportunistic when invading the cornea in FK.[1] Predisposing factors for developing FK in humans include advanced age, trauma (≤ 89% of cases) especially with vegetative foreign bodies, workers in rural or agricultural areas, immunosuppression, and past antibiotic, antifungal, or steroid use.[3, 4, 6] Mechanisms of fungal invasion and virulence have been extensively studied, including the requirement for transition from yeast to hyphal forms with *Candida*, expression of specialized proteins, such as adhesins and invasins on the cell surface, and development of biofilms.[7] Many of these virulence mechanisms represent areas of scientific investigation for developing new antifungal compounds or methods to prevent fungal invasion.[8, 9]

Identification of fungi as a possible causative organism of keratitis has traditionally been evaluated using direct cytological smears and the gold standard of culture and morphological-based identification.[2] Cultures reliably differentiate *Aspergillus*, *Candida*, and *Fusarium*, but due to the large degree of morphological variability at various developmental stages of growth, this traditional mycological classification approach does not provide consistent or discriminatory resolution to the species or genotype (lineage) level for identifying pathogenic fungal species known to infect the cornea.[2] Fungal molecular phylogenetic studies further define evolutionary lineages of fungi (i.e., a group of organisms that consists of all descendants of a common ancestor) that are animal and human pathogens beyond culture and routine identification techniques.[10–13] The ocular pathogens classified as *Fusarium*, for example, do not represent a single species but rather are members of a diverse species complex consisting of at least 18 phylogenetically distinct species.[10, 13] Species may exhibit differences in disease aggressiveness (e.g., corneal invasion and virulence) and susceptibility to antifungal medications, which if identified, could dramatically improve FK management since corneal ulcers are currently treated empirically routinely without susceptibility data.[6] Precise genotypic identification of FK etiological agents may also improve understanding of the environmental reservoir of each fungal species and epidemiology.[12] Molecular phylogenetic analysis and placement of fungal organisms causing FK is critical for diagnosis, therapy, particularly when correlated with disease outcome and prognostic aspects.[2]

Fungal keratitis is the most common cause of blindness in horses of the Southeastern USA and is a widespread disease in horses from all states east of the Rocky Mountains.[14–18] Similar to human keratitis, the most common causative organisms of FK in horses are the filamentous fungi, *Aspergillus* and *Fusarium*.[19] Clinically, FK in horses is also similar to human FK with characteristic diagnostic criteria of a raised corneal ulcer with a feathery border, satellite lesions, and secondary uveitis with hypopyon.[6, 16, 18] Once FK develops, current treatment is the same for all cases, regardless of fungal species, and greater than 50% of horses with FK do not respond to medical therapy and either require surgical repair or enucleation.[16] The similarity between human and equine FK suggests that there is high value in studying this naturally-occurring model of FK using molecular phylogenetic studies to predict aggressiveness and virulence of specific FK causative organisms and to select effective antifungal therapies.

The purpose of this study was to better understand the pathogenesis and treatment of FK by associating antifungal susceptibility and multi-locus sequence-based fungal identification with clinical outcome of a naturally occurring model of FK in horses.

## Methods

### Animals, disease assessment, and sample collection

Horses that were presented with FK to the ophthalmology service at North Carolina State University or Auburn University, confirmed through hyphae identified on wet mount cytological analysis with light microscopy, had culture samples collected from the clinically infected eye prior to initiating antifungal therapy. Following informed consent, samples were collected (using a sterile rayon swab or handle end of a sterile surgical blade) directly from the FK lesion. Samples were immediately plated using C-shaped streaks on Sabouraud dextrose agar (SDA) and trypticase soy agar with 5% sheep blood (CBA) and maintained at 25°C and 37°C for growth and microbiological identification. Signalment (age, breed and sex) and historical treatment and health information were also collected from each patient. Horses were treated with standard of care topical, subconjunctival, and/or systemic antifungal medications.[20] If medical therapy (MT) did not resolve the FK, then a surgical therapy (ST) such as a superficial keratectomy, keratectomy, conjunctival graft, or penetrating keratotomy was considered.[21, 22] Advanced disease, severe discomfort, or perforation of the eye usually resulted in enucleation (E).

### Fungal culture and identification

Inoculated SDA and CBA plates from the clinic were incubated and evaluated per standard operating procedures of the North Carolina State University Microbiology & Molecular Diagnostics Laboratory. Plates were incubated for up to 21 days, and evaluated biweekly for evidence of fungal growth. Initial fungal identification was performed based on examination of colony morphology and microscopic characteristics including shape and size of conidia, filamentous hyphae, chlamydospores, and conidiogenous cells following staining with lactophenol cotton blue.[1]

### DNA extraction, amplification and multi-locus sequencing

All fungi were sub-cultured onto Potato Dextrose Agar (PDA) to ensure cultures were pure and grown at 30°C for seven days in the dark. Mycelia of *Fusarium* spp. were harvested by straining through cheesecloth, lyophilized for 3 days, and stored at -80°C until DNA extraction. For cultures with characteristics of *Aspergillus*, conidia were harvested from the plates of PDA by flushing with 0.05% Triton X-100 and transferring the conidial suspension into a 2 mL Eppendorf tube. Tubes were stored at -20°C until DNA extraction. DNA was extracted using MOBIO UltraClean Kit protocol for *Aspergillus* and DNeasy Plant Mini Kit for *Fusarium*, following manufacturer's recommendations.

Multi-locus sequence typing (MLST) was performed with species-specific oligonucleotide primers (S1 Table) to identify species and evolutionary lineages. Initially, DNA for all isolates were amplified and sequenced with fungal-specific nuclear ribosomal internal transcribed spacer (ITS1) and the nuclear large-subunit rRNA (LR3) primers [23] to tentatively identify each fungus to genus/species level. Isolates of *Aspergillus flavus* were further genotyped using six loci: two aflatoxin cluster regions (*aflM/alfN* and *aflW/aflX*) and four non-cluster regions (*amdS*, *trpC*, *mfs*, and *MAT*) that provide resolution of specific *A*. *flavus* evolutionary lineages (IA, IB and IC) [24] and subspecies (*A*. *oryzae*).[25, 26] The *MAT1-1*and *MAT1-2* mating type genes in *A*. *flavus* were determined using oligonucleotide primers and methods described previously.[27] Isolates putatively identified as members of the *Fusarium solani* species complex were further genotyped using a portion of the DNA-directed RNA polymerase subunit 1 (*RPB1*) gene and two segments of the *RPB2* gene that were previously reported to provide resolution of *Fusarium* strains recovered from equine FK infected eyes.[13] See S1 Table for sequences of PCR primers used for multi-locus typing of *A*. *flavus* and *F*. *solani*. All samples were sequenced with forward primers with the exception of ITS1-LR3, which were sequenced with both forward and reverse primers (underlined in S1 Table). PCR master mix corresponding to each genus was made using Apex 2.0X Taq RED Master Mix, primers, and water. Each reaction contained 24 μL of master mix and 2 μL of DNA (1–3 ng/μL). All reactions were run in an Eppendorf Mastercycler ep Gradient S Thermocycler (Eppendorf, Hamburg, Germany) using cycling conditions presented in S2 Table. Amplified DNA products were subjected to electrophoresis in a 1.5% agarose gel with ethidium bromide to verify product size. Amplified PCR products were submitted for cleanup and Sanger sequencing at the North Carolina State University Genomic Sciences Laboratory.

### Phylogenetic placement and species identification

Sequences were examined in Sequencher version 5.4.6 (Gene Codes Corporation, Ann Arbor, MI). Ends were trimmed using default parameters to create unaligned FASTA sequence files for each locus. The Tree-Based Alignment Selector (T-BAS) toolkit v. 2.1 was used to integrate phylogenetic and taxonomic information, DNA sequence alignments, and clinical metadata, and to perform BLAST and phylogenetic placement of query FASTA sequences in the context of a predetermined reference tree.[28] BLASTn similarity searches of ITS sequences against the UNITE fungal database (Release 7, http://unite.ut.ee/index.php) [29] provided preliminary identification at the genus/species level. This was further corroborated with two-locus (ITS and LSU) likelihood-based placement on the published fungal [30] and Pezizomycotina [28] reference trees using the Evolutionary Placement Algorithm (EPA) in RAxML version 8 [31] accessible through the RESTful services at CIPRES.[32] Published reference trees, voucher information and multiple sequence alignments for *Aspergillus* section *Flavi* [25] and the *Fusarium solani* species complex [13] were imported into T-BAS v2.1 for reference-guided alignment and placement. This involves aligning query sequences for each locus to the homologous reference sequence alignment using MAFFT [33] and then running EPA on the newly extended multiple sequence alignments. A likelihood weight greater than 0.96 was used for identifying the nearest matching reference species, evolutionary lineage or MLST. Likelihood weights less than 0.5 indicate a weak match to the reference taxa and this could result in multiple equally probable or incorrect placements. In this case, MLSTs were determined directly for query isolates by collapsing multi-locus sequence alignments using SNAP Map [34] in the Mobyle SNAP Workbench. [35, 36]

### Assessment of antifungal minimum inhibitory concentration (MIC)

*In vitro* fungal susceptibility to voriconazole (VRC), natamycin (NAT), fluconazole (FLC), thiabendazole (THB), and terbinafine (TRB) were assayed in 96-well microplates using a modified protocol of the Clinical and Laboratory Standards Institute (CLSI) broth microdilution method (M38-A2 protocol) for filamentous fungi.[37] Moxifloxacin (MXF) was included as an antibacterial control. Antimicrobial compounds represented analytical grade formulations obtained from Sigma-Aldrich (St. Louis, MO) and were diluted with DMSO as a carrier agent. Agents were added to wells in 1 μl aliquots; the final concentration of the DMSO carrier was 0.5%. Each antimicrobial was tested in a 5x dilution series (0.01, 0.05, 0.25, 1.25, 6.25, 31, 70, 156 μg/ml) with 70 μg/ml inserted between 31 and 156 μg/ml and also in a 2x dilution series (0.063, 0.125, 0.25, 0.5, 1, 2, 4, 8, 16, 32 μg/ml) to refine the MIC determine within the middle part of the 5x dilution range. All isolates were evaluated in duplicate. Control wells included untreated wells and wells treated only with the DMSO carrier. None of the DMSO control wells showed inhibition of fungal growth. To avoid edge effects on treated wells, all edge wells were untreated. The volume of 50% Potato Dextrose Broth (PDB) in each well was 200 μl.

Isolates were cultured on PDA (Difco) and incubated at 30°C for seven days. Plates were flooded with 50% PDB (Difco) and filtered through cheesecloth to prepare conidial suspensions. Conidia concentrations were calculated using a hemocytometer and adjusted based on fungal genus (*Aspergillus* adjusted to 200,000 conidia/mL; *Fusarium* adjusted to 40,000 conidia/mL). Fifty μl of inoculum was delivered per well resulting in 10,000 conidia per well for *Aspergillus* and 2000 conidia per well for *Fusarium*. Microplates were incubated in the dark at 30°C for 72 hours. Minimum inhibitory concentrations (MICs), defined as the lowest concentration of an antifungal agent that substantially inhibits fungal growth, [37] were determined visually for each isolate using a magnified reading mirror.

### Data and statistical analysis

Associations among isolate, species, evolutionary lineage, mating type, signalment, disease type, and outcome (response to medical therapy, surgical therapy or enucleation) were evaluated using Wilcoxon signed rank and Fischer Exact tests. Associations between MIC values (using the lower MIC value of a range) and isolate, species, evolutionary lineage, mating type, signalment, disease type or outcome were determine using ANOVA, student t test, and Tukey’s post hoc analysis for multiple comparisons. Differences were considered significant at *p* ≤ 0.05 and all probabilities and results were calculated using computerized statistical software (JMP Pro, v. 13.2; SAS Inc., Cary, NC, USA). Additional statistical analyses of the MIC values were conducted as follows. First, median MIC values for each unique isolate–antifungal combination were calculated so that each isolate would be given equal weight. Then using Minitab 18, State College, PA, USA) ANOVA analyses were conducted. If the effect of interest (e.g. Isolate), was significant at the *p* ≤ 0.05 level, then Tukey mean separation was conducted with α = 0.05.

### Ethics statement

Animal use in this study adhered to the Association for Research in Vision and Ophthalmology Statement for use of animals in ophthalmic and vision research. Additionally, this study was approved and monitored by the North Carolina State University Institutional Care and Use Committee (IACUC) (Protocol approval # #12-013-O) and NC State Veterinary Hospital Board. The use of animals in research at NC State University is governed by institutional policy and at least two US federal statutes, including The Animal Welfare Act (Public Law 89–544, 1966, as amended [P.L. 91–579, P.L. 94–279, and P.L. 99–198]) and The Health Research Extension Act (P.L. 99–158, 1985, “Animals in Research”).

## Results

### Association between fungi isolated and clinical outcome

**Fig 1:**
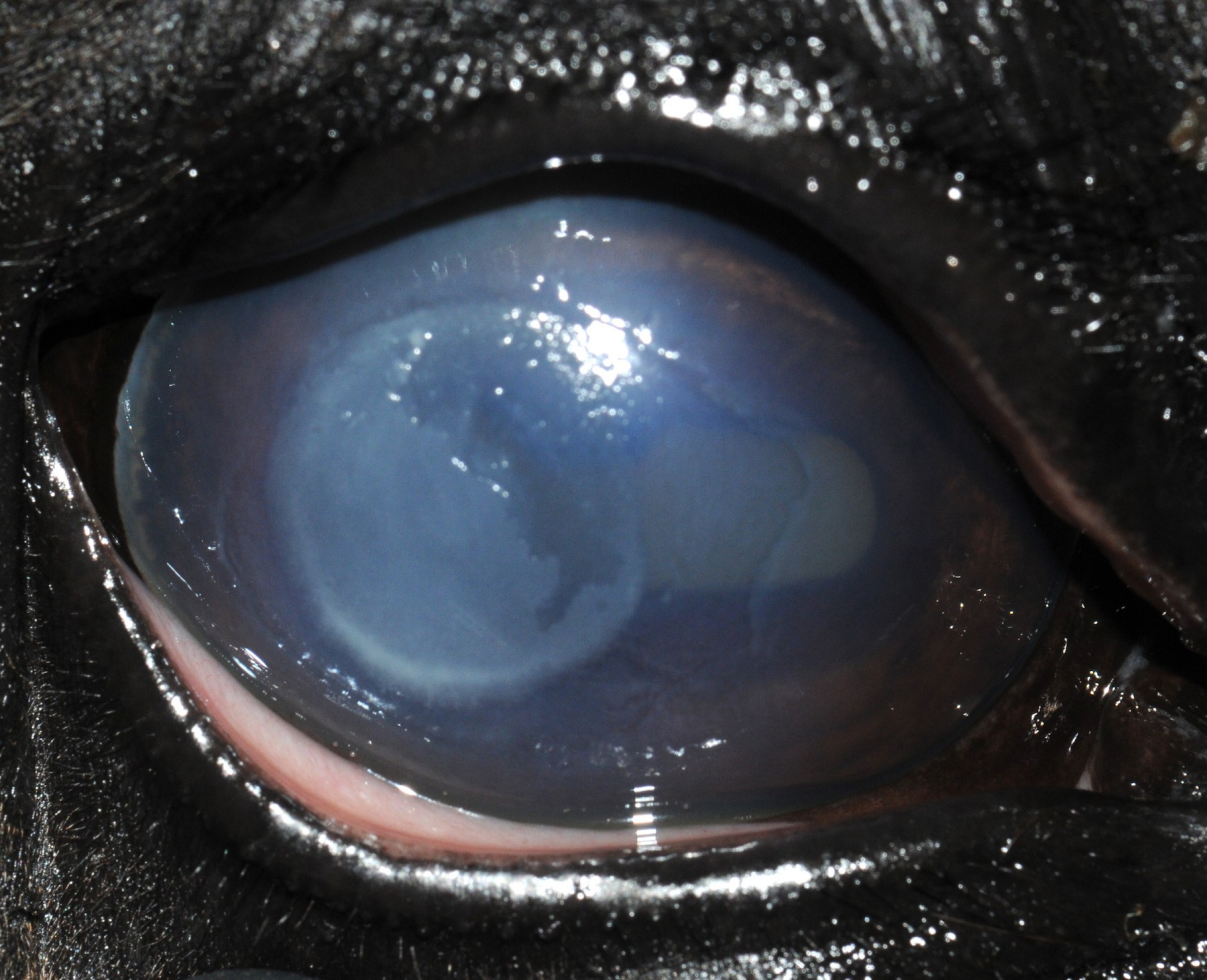
Superficial keratitis in a 24-year-old Thoroughbred horse (Horse #16) where *Aspergillus fumigatus* was isolated. This horse’s keratitis eventually healed following surgical keratectomy.

**Fig 2:**
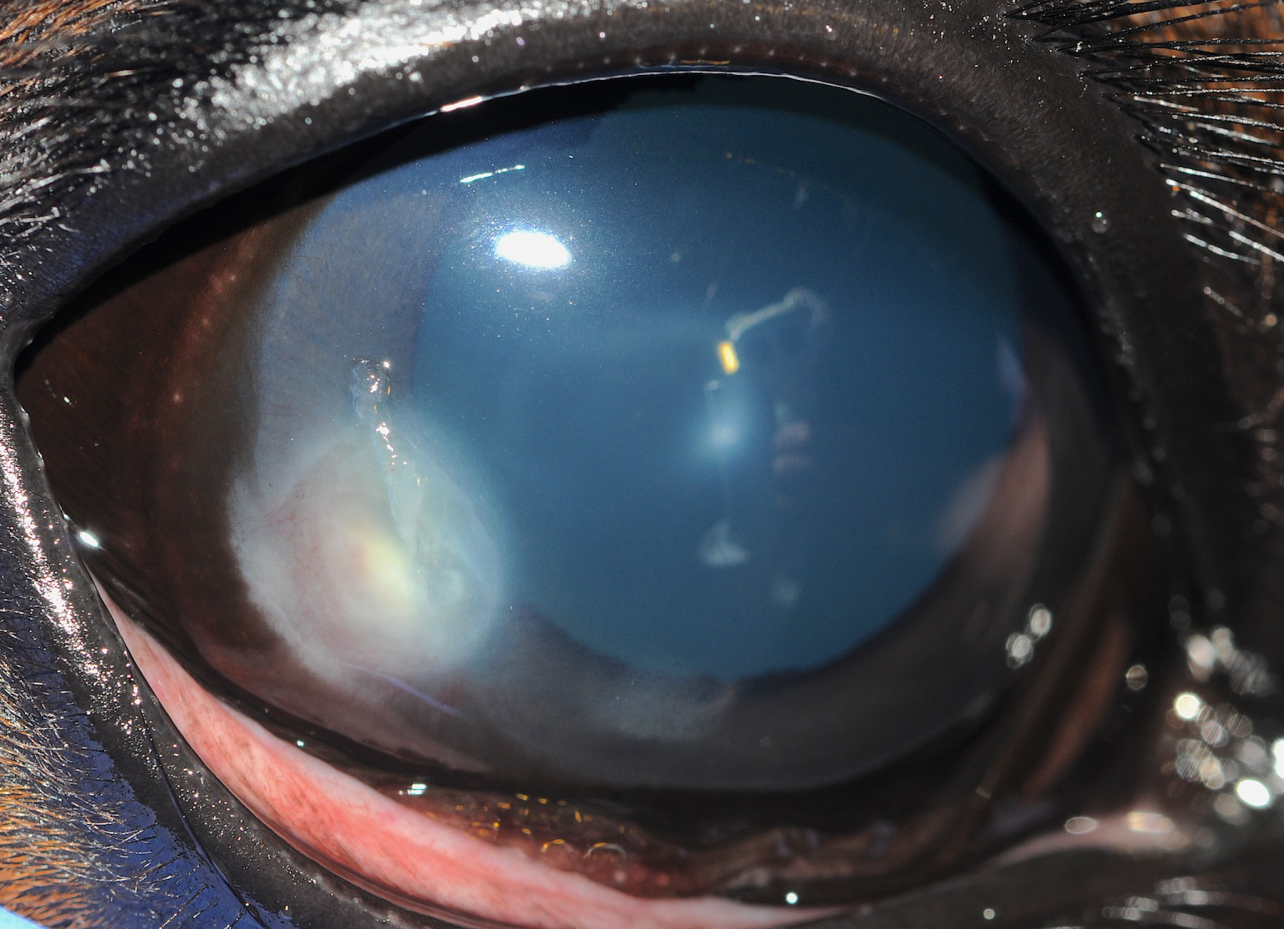
Stromal keratitis in a 6-month-old Holsteiner horse (Horse #29) where *Fusarium falciforme* was isolated. This horse’s keratitis healed with medical therapy consisting of topical voriconazole and natamycin.

Data and samples from 32 horses with fungal keratitis (FK) were evaluated. There were 15 breeds of horses affected with FK in this study, the most common of which mirrored the clinical population and included eight Quarter horses, six Thoroughbred, three Holsteiners, and three Tennessee walking horses. All horses were from the Southeastern US, with ~81% (26/32) from North Carolina. There were 22 males and 10 females, with a mean age of 14.0 years with a range of 0.6 to 36 years of age. The disease affected 18 right eyes and 14 left eyes with 13 eyes diagnosed with superficial FK (Fig 1) and 19 eyes presented with stromal FK (Fig 2). Outcome of the 32 eyes included eight (25%) that healed with medical therapy (MT), 12 (37.5%) that healed with surgical therapy (ST), and 12 (37.5%) that were either enucleated or the horse was euthanized because of severe FK (E). There was no significant association with outcome when evaluating horse breed, sex, age, eye affected, or type of corneal lesion (Tables 1 and 2).

**Table 1:** Signalment, type of corneal disease, outcome, bacteriological result, and fungal species metadata for equine fungal keratitis patients.

Patient #	Breed	Sex	Age at diagnosis (years)	City/State of Origin	Type of corneal disease	Fungal Species*	MLST** (Lineage)	Mating Type	Bacteriology Result	Outcome
1	Thoroughbred	MC	20	Cary, NC	Ulcerative–superficial	*Aspergillus flavus*	AF1 (IB)	*MAT1-1*	No growth	Healed with surgery
2	Paint Horse	MC	13	Eastover, SC	Ulcerative–stromal	*Aspergillus flavus*	AF1 (IB)	*MAT1-1*	No growth	Enucleation
3	Saddlebred	MC	15	Winston-Salem, NC	Ulcerative–stromal	*Aspergillus flavus*	AF2 (IB)	*MAT1-2*	No growth	Enucleation
4	Quarter Horse	MC	17	Raleigh, NC	Ulcerative—superficial	*Aspergillus flavus*	AF2 (IB)	*MAT1-2*	No growth	Enucleation
5	Pony	MC	22	Apex, NC	Ulcerative—stromal	*Aspergillus flavus*	AF2 (IB)	*MAT1-2*	No growth	Healed with medical therapy
6	Walking Horse	MC	17	Southern Pines, NC	Ulcerative—stromal	*Aspergillus flavus*	AF3 (IB)	*MAT1-2*	No growth	Healed with surgery
7	Fox Trotter	MC	10	Mount Olive, NC	Ulcerative—superficial	*Aspergillus flavus*	AF1 (IB)	*MAT1-1*	No growth	Healed with surgery
8	Thoroughbred	F	21	Roanoke, VA	Ulcerative—stromal	*Aspergillus flavus*	AF4 (IC)	*MAT1-2*	No growth	Healed with surgery
9	Walking Horse	MC	11	Marshville, NC	Ulcerative—stromal	*Aspergillus flavus*	AF5 (IC)	*MAT1-2*	No growth	Healed with surgery
10	Holsteiner	MC	7	Aberdeen, NC	Ulcerative—stromal	*Aspergillus flavus*	AF6 (IC)	*MAT1-1*	*Staphylococcus* sp.	Healed with surgery
11	Morgan	MC	10	Mooresville, NC	Ulcerative—superficial	*Aspergillus flavus*	AF7 (IC)	*MAT1-1*	*Kocuria rosea*	Enucleation
12	Quarter Horse	MC	22	Aberdeen, NC	Ulcerative—superficial	*Aspergillus flavus*	AF8 (IC)	*MAT1-2*	No growth	Enucleation
13	Quarter Horse	MC	14	Wake Forest, NC	Ulcerative—superficial	*Aspergillus flavus*	AF9 (IC)	*MAT1-1*	No growth	Healed with surgery
14	Thoroughbred	MC	2	Ocala, FL	Ulcerative—stromal	*Aspergillus flavus*	AF8 (IC)	*MAT1-2*	No growth	Enucleation
15	Quarter Horse	F	12	Birmingham, AL	Ulcerative—superficial	*Aspergillus flavus*	AF10 (IA)	*MAT1-1*	*Streptococcus equisimilis*	Healed with medical therapy
16	Thoroughbred	F	20	Southern Pines, NC	Ulcerative—superficial	*Aspergillus fumigatus*	n.d.	n.d.	No growth	Healed with surgery
17	Arabian	F	15	Hillsborough, NC	Ulcerative—stromal	*Aspergillus fumigatus*	n.d.	n.d.	No growth	Healed with surgery
18	Saddlebred	F	12	Colfax, NC	Ulcerative—superficial	*Aspergillus fumigatus*	n.d.	*MAT1-2*	No growth	Euthanasia
19	Thoroughbred	F	15	Oriental, NC	Ulcerative—stromal	*Fusarium falciforme*	FF1 (4dddd)	n.d.	No growth	Healed with surgery
20	Quarter Horse	MC	37	Ashboro, NC	Ulcerative—stromal	*Fusarium falciforme*	FF2 (4dddd, 4gggg)	n.d.	*Bacillus* spp.	Enucleation
21	Holsteiner	M	0.6	Midland, NC	Ulcerative—stromal	*Fusarium falciforme*	FF3 (4eee)	n.d.	No growth	Healed with medical therapy
22	Walking Horse	MC	17	Southern Pines, NC	Ulcerative—stromal	*Fusarium falciforme*	FF3 (4eee)	n.d.	No growth	Healed with surgery
23	Dutch Warmblood	F	15	Williamsburg, VA	Ulcerative—stromal	*Fusarium falciforme*	FF4 (4eeee, 4uuu)	n.d.	*Streptococcus zooepidemicus*	Healed with medical therapy
24	Selle Francais	MC	16	Davidson, NC	Ulcerative—stromal	*Fusarium falciforme*	FF5 (4hhhh)	n.d.	No growth	Enucleation
25	Quarter Horse	MC	11	Advance, NC	Ulcerative—stromal	*Fusarium falciforme*	FF6 (4hhhh, 4ffff)	n.d.	No growth	Healed with medical therapy
26	Warmblood	MC	10	Hillsborough, NC	Ulcerative—superficial	*Fusarium falciforme*	FF7 (4hhhh, 4ffff)	n.d.	No growth	Enucleation
27	Warmblood	F	14	Reidsville, NC	Ulcerative—stromal	*Fusarium falciforme*	FF8 (4hhhh, 4ffff)	n.d.	No growth	Enucleation
28	Percheron	MC	22	Sedley, VA	Ulcerative—stromal	*Fusarium keratoplasticum*	FK1 (2u)	n.d.	No growth	Enucleation
29	Holsteiner	M	7	Midland, NC	Ulcerative—stromal	*Fusarium proliferatum*	FP1	n.d.	No growth	Healed with medical therapy
30	Quarter Horse	F	5	Warsaw, NC	Ulcerative—superficial	*Mucor* sp.	n.d.	n.d.	*Staphylococcus aureus*	Healed with medical therapy
31	Quarter Horse	F	11	Summerton, SC	Ulcerative—stromal	*Byssochlamys* sp.	n.d.	n.d.	No growth	Healed with medical therapy
32	Thoroughbred	MC	11	Wilmington, NC	Ulcerative—superficial	*Exserohilum* sp.	n.d.	n.d.	*Bacillus* spp.	Healed with surgery

n.d. = Not Determined

*Classification to species level was based on multi-locus phylogenetic placement.

**Multi-locus sequence type (MLST) designations are labeled with the first two uppercase letters for the species (AF = *A*. *flavus*; FF = *F*. *falciforme*; FK = *Fusarium keratoplasticum*; and FP = *Fusarium proliferatum*) followed by a number for the unique haplotype within each species. In parentheses are lineage or species haplotype designations derived from reference trees used for phylogenetic placements. In *A*. *flavus*, lineage membership (IA, IB, or IC) is from Moore et al 2017 (25). In *Fusarium*, species haplotypes are shown instead of lineage and are from O’Donnell et al. 2016 (13), where species are designated with Arabic numerals (2 = *F*. *keratoplasticum*; and 4 = *F*. *falciforme*) followed by lowercase letters to represent unique haplotypes within each species (e.g. 4dddd and 4gggg represent different multi-locus haplotypes).

**Table 2:** Summary table—Genetic lineage haplotypes, species haplotypes and clinical outcomes in fungal keratitis.

Fungal identification	Lineage haplotypes	Clinical type (n)	Outcome (n)*
***Aspergillus spp*.**	*A*. *flavus* lineage IB		
	AF1	Superficial (2); Stromal (1)	HS (2) E (1)
	AF2	Superficial (1); Stromal (2)	HM (1) E (2)
	AF3	Stromal (1)	HS (1)
	*A*. *flavus* lineage IC		
	AF4	Stromal (1)	HS (1)
	AF5	Stromal (1)	HS (1)
	AF6	Stromal (1)	HS (1)
	AF7	Superficial (1)	E (1)
	AF8	Superficial (1); Stromal (1)	E (2)
	AF9	Superficial (1)	HS (1)
	*A*. *flavus* lineage IA		
	AF10	Superficial (1)	HS (1)
	*A*. *fumigatus*	Superficial (2); Stromal (1)	E (1)^a^ HS (2)
	**Total**	**Superficial (9); Stromal (9)**	**HM (1) HS (10) E (6) E (1)**^**a**^
***Fusarium spp*.**	**Species haplotypes:**		
	*F*. *falciforme* FF1	Stromal (1)	HS (1)
	*F*. *falciforme* FF2	Stromal (1)	E (1)
	*F*. *falciforme* FF3	Stromal (2)	HM (1) HS (1)
	*F*. *falciforme* FF4	Stromal (1)	HM (1)
	*F*. *falciforme* FF5	Stromal (1)	E (1)
	*F*. *falciforme* FF6	Stromal (1)	HM (1)
	*F*. *falciforme* FF7	Superficial (1)	E (1)
	*F*. *falciforme* FF8	Stromal (1)	E (1)
	*F*. *proliferatum* FP1	Stromal (1)	HM (1)
	*F*. *keratoplasticum* FK1	Stromal (1)	E (1)
	**Total**	**Superficial (1); Stromal (10)**^**1**^	**HM (4) HS (2) E (5)**
**Other**	*Mucor circinelloides*	Superficial (1)	HM (1)
	*Byssochlamys* sp.	Stromal (1)	HM (1)
	*Exserohilum* sp.	Superficial (1)	HS (2)
	**Total**	**Superficial (2); Stromal (1)**	**HM (2) HS (1) E (0)**
	**Total Isolates**	**Superficial (12); Stromal (20)**	**HM (7) HS (13) E (11) E (1)**^**a**^

*HM–healed with medical treatment only

^a^ Euthanasia instead of enucleation

HS- healed with surgical intervention. E–enucleated

^1^*Fusarium* sp. fungal keratitis significantly more likely to be associated with stromal keratitis (Fishers Exact test, *p* = 0.045)

On routine fungal culture, characteristic microconidia (oval and 1–2 cells) and macroconidia (curved (falcate) and >2 cells) and chlamydospores typical of *Fusarium* and oval chains of conidia attached to phialides and metulae arising for vesicle typical of *Aspergillus* were observed for 90.6% (29/32) of the cultures. The most common fungi isolated based on morphological and DNA analysis in horses with FK were species of *Aspergillus* (18 of 32 [50%]) and *Fusarium* (11 of 32 [34%]). In addition, three other fungal species (*Byssochlamys*, *Mucor*, and *Exserohilum*) were identified and associated with equine FK (Table 1). Bacterial outcomes were reported for six horses with FK (18.8%) and consisted of species of *Bacillus* (1), *Staphylococcus* (2), *Streptococcus* (2) and *Kocuria rosea* (1) (Table 1). There were no statistical associations among fungal species, type of corneal lesion, presence of bacterial co-infection, or patient outcome (Tables 1 and 2). However, species of *Fusarium* sampled and cultured from FK horses were significantly (*p* = 0.045; Fisher’s Exact test) more likely to be associated with stromal keratitis.

Horse eyes infected with *Fusarium* were significantly (Chi-Square *p* = 0.04) more likely to heal with medical therapy than eyes infected with *Aspergillus*. But the enucleation level was essentially the same whether the eye was infected with *Aspergillus* or *Fusarium* (*p* = 0.88) because of improved healing with surgery for eyes infected with *Aspergillus*.

To delimit species in *Fusarium*, sequences were examined using multi-locus EPA placement on the reference tree published by O'Donnell et al. [13] *Fusarium* multi-locus haplotypes were based on collapsing of concatenated *RPB1* and *RPB2* sequence alignments. In our naming convention, multi-locus haplotypes are labeled with the first two uppercase letters for the species (e.g., FF = *F*. *falciforme*) followed by a number for the unique haplotype within each species. Of the *Fusarium* species isolated from equine FK, 10/11 (91%) samples belonged to the *Fusarium solani* species complex (FSSC) (i.e., nine isolates of *Fusarium falciforme* and one isolate of *Fusarium keratoplasticum*). An additional isolate of *Fusarium proliferatum* belonging to the *Fusarium fujikuroi* species complex (FFSC) was also sampled from equine FK. FSSC haplotypes were labeled FF1–8, FP1 and FK 1 (Tables 1 and 2). *Fusarium* species, haplotypes, isolates, or presence of bacterial co-infection was not significantly associated with lesion type or FK outcome.

To determine lineage membership for species identified as *Aspergillus flavus*, sequences were examined using multi-locus EPA placement on the *Aspergillus* section *Flavi* reference tree. [25] *Aspergillus* lineage haplotypes were based on *aflM/alfN*, *aflW/aflX*, *amdS*, *trpC*, *mfs*, and *MAT*. Of the species of *Aspergillus* isolated from equine FK, 15 were classified as *Aspergillus flavus*, seven of which were lineage IB, seven belonging to lineage IC and one from lineage IA. Three isolates were classified as *Aspergillus fumigatus* (Tables 1 and 2). *Aspergillus flavus* lineage haplotypes were labeled AF1–10 (Tables 1 and 2). *Aspergillus* species, evolutionary lineage and haplotypes, or presence of co-infection was not significantly associated with lesion type or outcome of FK (Tables 1 and 2).

Three other fungi isolated from equine FK included species of *Mucor*, *Byssochlamys*, and *Exserohilum*. *Mucor* and *Exserohilum spp*. both had bacterial co-infections, however, all three patients healed, two with medical treatment only, and one with surgical treatment (Table 1). Overall, these outcome results were more favorable than FK with *Aspergillus spp*. (2 HM; 9 HS; 7 E) or *Fusarium spp*. (4 HM; 2 HS; 5 E) (Tables 1 and 2).

### Association between *in vitro* antifungal susceptibility and fungal taxonomy

**Fig 3:**
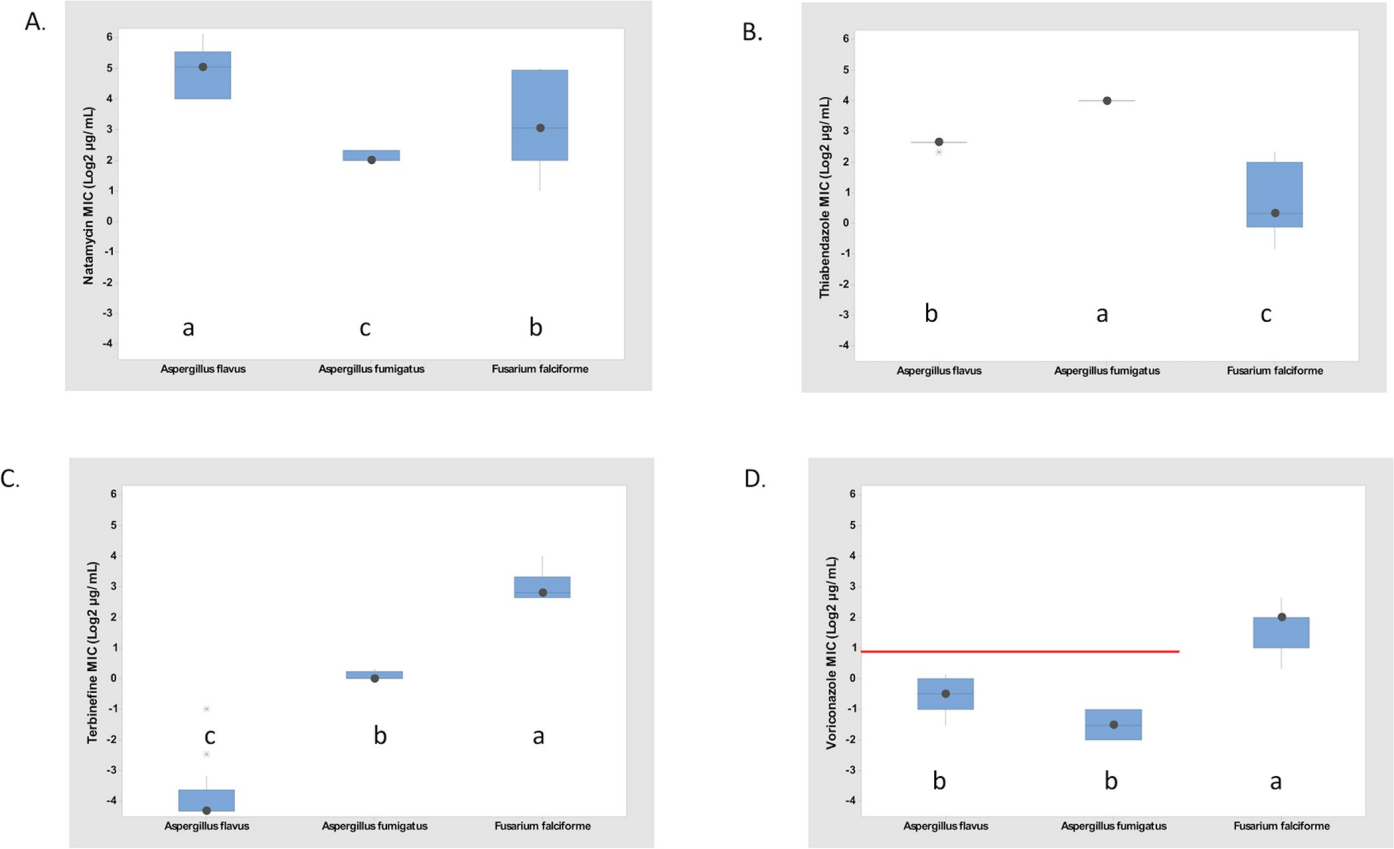
Fungal species boxplots of isolates sampled from equine fungal keratitis. A. Natamycin. B. Thiabendazole. C. Terbinafine. and D. Voriconazole. Minimal inhibitory concentration (MIC) values converted to log base 2 in parallel to the 2x dose steps used. ANOVA 2-factor Analysis: P values were all < 0.001 for fungus and antifungal main effects and for fungus x antifungal interaction. Mean separation of the fungus x antifungal interaction: Tukey mean with α = 0.05. Different letters indicate significant differences, CLSI susceptibility working breakpoint for voriconazole for *Aspergillus* is ≤1 μg/mL (red line). No breakpoints are available for natamycin, thiabendazole and terbinafine. Number of isolates: *Aspergillus flavus*: n = 13; *Aspergillus fumigatus*: n = 5; and *Fusarium falciforme*: n = 10.

*In vitro* antifungal susceptibility of VRC, NAT, FLC, THB, TRB, and MXF (as a negative control) was evaluated for isolates of *Aspergillus* and *Fusarium* from equine FK (Table 3; Fig 3). None of the fungal isolates were susceptible to MXF even at concentrations as high as 156 μg/ml. All *Fusarium* isolates and most *Aspergillus* isolates grew in the presence of FLC at concentrations as high as 156 μg /ml. There were no significant association in mean MIC values for FLC and MXF among isolates, species, evolutionary lineages, degree of corneal invasion, or disease outcome (Table 3).

**Table 3:** Minimal inhibitory concentrations (MIC) (μg/mL) of isolates from equine fungal keratitis.

Patient #	Type of corneal disease	Fungal Species	MLST* (Lineage)	Voriconazole MIC	Natamycin MIC	Fluconazole MIC	Thiabendazole MIC	Terbinafine MIC	Moxifloxacin MIC	Antifungal (s) used	Outcome
1	Ulcerative—superficial	*Aspergillus flavus*	AF1 (IB)	0.5	n.d.	>156	n.d.	n.d.	>156	Voriconazole, natamycin	Healed with surgery
2	Ulcerative—stromal	*Aspergillus flavus*	AF1 (IB)	0.5–1	16	≥156	6.25	0.05	>156	n.a.	Enucleation
3	Ulcerative—stromal	*Aspergillus flavus*	AF2 (IB)	0.5–1	32–70	≥156	6.25	0.05	>156	Voriconazole, fluconazole	Enucleation
4	Ulcerative—superficial	*Aspergillus flavus*	AF2 (IB)	0.25–1	16–70	≥156	6.25	0.05	n.d.	Voriconazole, amphotericin B	Enucleation
5	Ulcerative—stromal	*Aspergillus flavus*	AF2 (IB)	0.5–1	16	>156	6.25	0.05–0.25	n.d.	Voriconazole, fluconazole	Healed with medical therapy
6	Ulcerative—stromal	*Aspergillus flavus*	AF3 (IB)	0.5–1	16–70	≥156	6.25	0.05–1	>156	n.a.	Healed with surgery
7	Ulcerative—superficial	*Aspergillus flavus*	AF1 (IB)	0.5–1	≥32	>156	6.25	0.05–0.0625	n.d.	Voriconazole	Healed with surgery
8	Ulcerative—stromal	*Aspergillus flavus*	AF4 (IC)	0.05–0.26	16–70	≥156	4–6.25	0.05–0.25	>156	Voriconazole	Healed with surgery
9	Ulcerative—stromal	*Aspergillus flavus*	AF5 (IC)	1.25–4	70	>156	6.25–16	0.05–0.125	>156	n.a.	Healed with surgery
10	Ulcerative—stromal	*Aspergillus flavus*	AF6 (IC)	0.25–0.5	4–6.25	>156	16	1	n.d.	n.a.	Healed with surgery
11	Ulcerative—superficial	*Aspergillus flavus*	AF7 (IC)	1	32–70	>156	6.25	0.125–0.25	n.d.	Voriconazole	Enucleation
12	Ulcerative—superficial	*Aspergillus flavus*	AF8 (IC)	1–1.25	70	>156	6.25	0.0625	n.d.	None	Enucleation
13	Ulcerative—superficial	*Aspergillus flavus*	AF9 (IC)	1	32	>156	6.25	0.05	n.d.	Voriconazole	Healed with surgery
14	Ulcerative—stromal	*Aspergillus flavus*	AF8 (IC)	0.5–1	32–70	≥156	6.25	0.05	n.d.	Miconazole, Voriconazole, Amphotericin B	Enucleation
15	Ulcerative—superficial	*Aspergillus flavus*	AF10 (IA)	0.5–1	16–70	≥156	6.25	0.05–1	>156	Voriconazole, natamycin	Healed with medical therapy
16	Ulcerative—superficial	*Aspergillus fumigatus*	n.d.	0.25–0.5	4–6.25	>156	16	1	>156	Voriconazole	Healed with surgery
17	Ulcerative—stromal	*Aspergillus fumigatus*	n.d.	0.25–0.5	4–6.25	>156	16	1–1.25	>156	Voriconazole, natamycin	Healed with surgery
18	Ulcerative—superficial	*Aspergillus fumigatus*	n.d.	0.25–1.25	4	>156	16	1.25	>156	None	Euthanasia
19	Ulcerative—stromal	*Fusarium falciforme*	FF1 (4dddd)	2–6.25	4–32	>156	1.25	6.25–16	>156	n.a.	Healed with surgery
20	Ulcerative—stromal	*Fusarium falciforme*	FF2 (4dddd, 4gggg)	2	4–8	>156	1	6.25–16	>156	Voriconazole	Enucleation
21	Ulcerative—stromal	*Fusarium falciforme*	FF3 (4eee)	2–4	4–32	>156	4–6.25	6.25	>156	Voriconazole	Healed with medical therapy
22	Ulcerative—stromal	*Fusarium falciforme*	FF3 (4eee)	2–4	4–32	>156	4	6.25	>156	n.a.	Healed with surgery
23	Ulcerative—stromal	*Fusarium falciforme*	FF4 (4eeee, 4uuu)	4	4–70	>156	1.25	6.25–16	>156	Voriconazole	Healed with medical therapy
24	Ulcerative—stromal	*Fusarium falciforme*	FF5 (4hhhh)	1–4	4–32	>156	0.25–1.25	6.25–8	>156	Voriconazole	Enucleation
25	Ulcerative—stromal	*Fusarium falciforme*	FF6 (4hhhh, 4ffff)	6.25	4	>156	4	16	n.d.	Voriconazole, fluconazole	Healed with medical therapy
26	Ulcerative—superficial	*Fusarium falciforme*	FF7 (4hhhh, 4ffff)	1–4	0.125–32	>156	0.25–2	6.25–8	>156	Voriconazole, fluconazole	Enucleation
27	Ulcerative—stromal	*Fusarium falciforme*	FF8 (4hhhh, 4ffff)	1–6.25	1.25–32	>156	1–4	6.25	>156	Voriconazole, fluconazole	Enucleation
28	Ulcerative—stromal	*Fusarium keratoplasticum*	FK1 (2u)	4–6.25	6.25–32	>156	1.25–6.25	16	>156	n.a.	Enucleation
29	Ulcerative—stromal	*Fusarium proliferatum*	FP1	1.25–4	1.25–4	>156	8	1.25	>156	Voriconazole	Healed with medical therapy
30	Ulcerative—superficial	*Mucor* sp.	n.d.	>156	6.25	>156	n.d.	n.d.	>156	Voriconazole	Healed with medical therapy
31	Ulcerative—stromal	*Byssochlamys* sp.	n.d.	4	n.d.	>156	n.d.	n.d.	>156	Voriconazole, fluconazole	Healed with medical therapy
32	Ulcerative—superficial	*Exserohilum* sp.	n.d.	n.d.	n.d.	n.d.	n.d.	n.d.	n.d.	Voriconazole	Healed with surgery

n.d. = Not Determined. n.a. = Not Available

*See Table 1 for a description of MLST (Lineage

Minimal inhibitory concentration values for VRC ranged from 0.25 μg/ml (five isolates of *Aspergillus flavus*) to 6.25 μg/ml for four *Fusarium* isolates. An isolate of *Mucor* sp. had an MIC of >156 μg/ml for VRC. For NAT, MIC ranged from 0.125 μg/ml (one isolate of *Fusarium falciforme* [FF7]) to 32 μg/ml for five isolates of *A*. *flavus*. Minimal inhibitory concentration values for THB ranged from 0.25 μg/ml against two isolates of *Fusarium falciforme* to 16 μg/ml for an *A*. *flavus* and three *A*. *fumigatus* isolates. For TRB, MIC values ranged from 0.05 μg/ml from 11 isolates of *Aspergillus* spp. to 16 μg/ml for a *F*. *falciforme* and *F*. *keratoplasticum* isolate. (Table 3).

At the fungal genus level, there were highly significant differences in sensitivity of *Aspergillus* and *Fusarium* isolates for three compounds, VRC, THB, and TRB (p<0.001). *Aspergillus* was more sensitive to VRC and TRB than *Fusarium*; whereas *Fusarium* was more sensitive to THB than *Aspergillus* (Table 3, Fig 3). For NAT, the strong species effect within *Aspergillus* resulted in one *Aspergillus* species being more sensitive and one species being less sensitive than the *Fusarium* isolates which were intermediate in sensitivity between the two species of *Aspergillus*. Therefore, patterns of sensitivity to NAT have to be considered at the species level not at the genus level.

**Fig 4:**
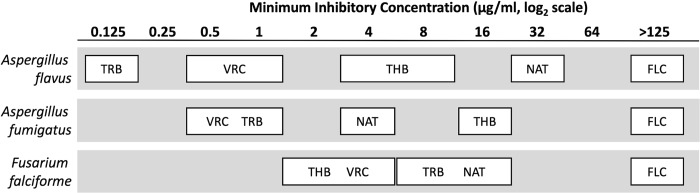
Minimal inhibitory concentration (MIC) comparisons among isolates from equine fungal keratitis. Antifungal agents within a box do not have significantly different MIC values, while antifungal agents in different boxes have significantly different MIC values. ANOVA, 2-factor. P < 0.001 for fungus x antifungal agent interaction. Mean separation: Tukey with α = 0.05. FLC = fluconazole, NAT = Natamycin, TRB = Terbinafine, THB = Thiabendazole, and VRC = Voriconazole.

At the fungal species level, there were significant (p<0.001) differences among species for four antifungal agents, VRC, THB, NAT, and TRB (Figs 3 and 4). Three species had multiple isolates and thus could be tested for MIC species differences: *Aspergillus flavus*, *A*. *fumigatus*, and *Fusarium falciforme*. *A*. *flavus* (mean MIC of 33.5 +/- SD 16.1 μg/ml) was less susceptible than *F*. *falciforme* (mean MIC of 14.4 +/- SD 12.4) and *A*. *fumigatus* (mean MIC of 4.4 +/- SD 0.5) to NAT. Both species of *Aspergillus* (with mean MIC of 0.10 +/- SD 0.13 μg/ml for *A*. *flavus* and 1.1 +/- SD 0.11 μg/ml for *A*. *fumigatus*) were more susceptible than *F*. *falciforme* (mean MIC of 8.5 +/- SD 3.1) to TRB. For VRC the two species of Aspergillus had mean MIC values of 0.7 +/- SD 0.3 μg/ml for *A*. *flavus* and 0.4 +/- SD 0.1 μg/ml for *A*. *fumigatus* and exhibited greater susceptibility than *F*. *falciforme* with a mean MIC of 3.4 +/- SD 1.5 μg/ml. In contrast, *Fusarium falciforme* (mean MIC of 2.1 +/- SD 1.6) was more susceptible to THB than both species of *Aspergillus* (mean MIC of 6.2 +/- SD 0.3 μg/ml for *A*. *flavus* and 16.0 +/- SD 0.0 μg/ml for *A*. *fumigatu*s) (Figs 3 and 4).

There were significant differences in susceptibility between the two species of *Aspergillus* for NAT, THB, and TRB. *A*. *fumigatus* was more susceptible to NAT than *A*. *flavus* (mean MIC of 4.4 +/- SD 0.5 μg/ml for *A*. *fumigatus* and 33.5 +/- SD 16.1 μg/ml for *A*. *flavus*) whereas *A*. *flavus* exhibited higher susceptibility to TBH and to TRB than *A*. *fumigatus* (TBH: mean MIC of 6.2 +/- SD 0.3 μg/ml for *A*. *flavus* and 16.0 +/- SD 0.0 μg/ml for *A*. *fumigatus*; TRB: mean MIC of 0.1 +/- SD 0.1 μg/ml for *A*. *flavus* and 1.1 +/- SD 0.1 μg/ml for *A*. *fumigatus*). These statistically significant species differences in antifungal agent susceptibility within *Aspergillus* demonstrate the importance of accurate identification of the causal fungal pathogen to the species level. In the case of *Fusarium*, it was not possible to evaluate interspecies differences because there was only one isolate of *F*. *keratoplasticum* and *F*. *proliferatum*. However, the pattern of TRB and THB MIC values between the single isolate of *F*. *proliferatum* and the 9 isolates of *F*. *falciforme* suggest that there may be interspecies susceptibility differences within *Fusarium*. The median TRB MIC value for *F*. *proliferatum* at 1.25 μg/ml was lower than the TRB MIC values for all 9 isolates of *F*. *falciforme*. The median THB MIC for *F*. *proliferatum* at 8 μg/ml was higher than the THB MIC values for all 9 isolates of *F*. *falciforme*.

There were no statistically significant differences in antifungal agent susceptibility between IB and IC lineages within *A*. *flavus* and among different lineages of *F*. *falciforme* in antifungal agent susceptibility to TRB, TBH and VRC. Lineage group FF6-7-8 of *F*. *falciforme* was more susceptible than lineages FF1, 2, 4, and 5 to NAT. Neither of these lineage groups of *F*. *falciforme* differed in susceptibility to the two isolates belonging to lineage FF3 which were classified as intermediate.

The antifungal used for treatment in these equine FK cases included most commonly topical voriconazole (n = 23/32), topical natamycin (n = 3/32), oral fluconazole (n = 7/23), and subconjunctival amphotericin B (n = 2/32) (Table 2). The selection and route of these antifungals was based on formulation availability and clinician preferences, and not on susceptibility testing. There was no correlation between *in vitro* sensitivity testing, antifungal used, and FK outcome (Table 3).

## Discussion

FK is a common and aggressive disease in horses. In this study, 25% of equine FK cases were resolved with medical therapy and over 37% of the patients had loss of the eye due to infection. To better understand the pathogenesis and treatment of this disease, we used multi-locus DNA sequence analysis to accurately determine fungal species and evolutionary lineages and to examine associations with *in vitro* antifungal agent susceptibility, and outcome of equine FK. Analogous to human patients, misidentification of causative agents of filamentous FK and use of inadequate therapy may lead to blindness. Therefore, species-level identification of putative pathogen and antifungal agent susceptibility of the causal fungi is important for successful FK therapy.[38]

In this study of 32 cases of FK in horses, filamentous fungi predominated: 56% of FK cases were associated with *Aspergillus* spp., 34% with *Fusarium* spp., and 3% were *Mucor* sp., *Byssochlamys* sp., or *Exserohilum* sp. Our results are consistent with previous reports using standard mycological culture techniques in horses where the occurrence and isolation of species of *Aspergillus* predominate in equine FK, with species of *Fusarium* sampled and isolated in a lower frequency than *Aspergillus*.[39–41] Associated fungal species in human FK vary, but similar to horses, filamentous fungi predominate. In most studies of human FK investigations, a slightly higher percentage of species of *Fusarium* (approximately 28–48%) is observed compared to species of *Aspergillus* (19–25%).[1,3,4] However, in a study from China, FK in humans were more commonly associated with *A*. *fumigatus* (65%)[1], while another study from south Florida demonstrated *A*. *flavus* (42%) as the most common fungal associate in human cases of FK,[6] suggesting a regional geographic difference in pathogenic fungal species in FK.

In both equine and human *Fusarium* FK, fungi most commonly isolated belong to the *F*. *solani* species complex (FSSC) (i.e., *Fusarium falciforme*, *Fusarium keratoplasticum* and *Fusarium* sp. FSSC 12). Gajjar *et al*.[6], Homa *et al*. [42] and Oechsler *et al*. [2] also found that FK *Fusarium* sampled from human eyes nested most commonly into the FSSC. For example, Gajjar *et al*. [6] used a single locus (ITS1 and 4 regions) for phylogenic analysis and placement and reported that all identified isolates of *Fusarium* placed into the FSSC. Homa *et al*. [42] conducted a two-locus (β-tubulin and elongation factor 1-α) and Oechsler *et al*. [2] a single locus (ITS) phylogenetic analyses of *Fusarium* collected from human eyes in India and South Florida, respectively, also demonstrated that 75–76% of *Fusarium* causing FK belonged to the FSSC. O’Donnell [13] described species of *Fusarium* isolated from a variety of veterinary sources and found that the most commonly sampled veterinary *Fusaria* were isolated from eyes of horses (31% of those reported). Furthermore, they deployed a three-locus phylogenetic analysis (*TEF*1, *RPB2*, and ITS) of 17 isolates of *Fusarium* sampled from 17 equine eyes, most of which were from the southeastern US. Similar to our results, O’Donnell reported 14 of 17 (82%) isolates sampled from an equine FK source belonged the FSSC and represented 12 genetically diverse strains/lineages.[13] In our study, 91% of equine *Fusarium* FK nested within the FSSC and represented nine genetically diverse strains/lineages. Only MLSTs from horse numbers 21, 22 and 29 had cumulative likelihood weights > 0.96 and are considered reliable placements within the FSSC*; F*. *falciforme* haplotype FF3 for patient 21 and 22 matched *F*. *falciforme* haplotype 4eee from equine eye (NRRL 54964); *F*. *proliferatum* FP1 for patient 29 matched rhinoceros horn (NRRL 54994) and equine eye (NRRL 62546); all other strains showed weak placements and hit multiple *F*. *falciforme* haplotypes as nearest siblings. It is common for members of the FSSC that share the same multi-locus haplotypes to cause infections in humans, animals and plants.[43] This is true also in *F*. *falciforme* which was reported as an emerging pathogen on lima bean in Brazil [44] and shares a most recent common ancestor with *F*. *falciforme* haplotypes in this study based on phylogenetic placement of a portion of the *RPB2* gene (data not shown). Updating the FSSC reference tree with these strains would increase phylogenetic and host diversity of *F*. *falciforme*, and improve resolution and reliability of future placements.

In our study, 15/18 (83%) of equine *Aspergillus* FK nested within the *A*. *flavus* clade, and included three genetically diverse lineages, IA, IB and IC. Only one *A*. *flavus* isolate belonged to IA and the other 14 strains were equally split between IB and IC, which is consistent with the frequency of IB and IC isolated from soil in agricultural fields.[25, 45] Interestingly, 10/14 (71%) of the *A*. *flavus* strains had *A*. *oryzae* as their nearest common ancestor in both lineages IB (7/7) and IC (3/7), supporting a close relationship between wild and domesticated *A*. *flavus* strains.[26, 46] Putative clonal lineages within IB (AF1) and IC (AF8) were associated with both superficial and stromal keratitis infections in different horses and states, suggesting that strains with close affinities to *A*. *flavus/A*. *oryzae* harbor characteristics (e.g. metabolites) that serve as effective conduits for equine FK disease. Three additional isolates of *Aspergillus* were identified as *A*. *fumigatus* (17%). Further differentiation of these strains is possible using mating types [47] and microsatellite markers [48] but we have limited information on evolutionary lineages in *A*. *fumigatus* from multi-locus DNA sequence data. There are fewer studies specifically evaluating the genetic diversity of *Aspergillus* in human FK.[49] However, in one study in India [49], fungi identified through multi-locus sequence analysis (ITS1-5.8S-ITS2, calmodulin, and β-tubulin) were similar to what we found in horses where 75% of human FK aspergillosis were identified as *A*. *flavus* and 12% were *A*. *fumigatus*.

Although *A*. *flavus/A*. *oryzae* and *F*. *falciforme* were recovered predominantly from equine FK infected eyes, species, haplotypes, isolates, or evolutionary lineage of *Aspergillus* or *Fusarium* were not significantly associated with lesion type or FK outcome in horse eyes in this study. This suggests that FK disease severity or virulence are complex phenotypes determined by multiple factors that are not closely linked to multi-locus markers examined in this study. Additional factors such as initiating injury (the type and nature of injury is typically unknown in horses), delay of owners of horses to seek treatment, and variable treatment prior to examination may determine severity of infection and outcome in equine FK. However, in this study we demonstrated that *Fusarium* species sampled and cultured from FK horses were significantly more likely to be associated with stromal keratitis compared to *Aspergillus*.

Although there was no statistical association among antifungal agent susceptibility and disease severity or outcome, significant differences in susceptibility was observed at the fungal genus, species, and evolutionary lineage levels. Most notably, at the fungal genus level, *Aspergillus* was more susceptible to VRC and TRB than *Fusarium*; whereas *Fusarium* was more susceptible to THB than *Aspergillus*. At the species level, *A*. *flavus* was statistically less sensitive to NAT than *F*. *falciforme* and *A*. *fumigatus*. Both species of *Aspergillus* were more susceptible to TRB than *F*. *falciforme* and the two species of *Aspergillus* were more susceptible to VRC than *F*. *falciforme*. In contrast, *Fusarium falciform*e was more susceptible to THB than were both *Aspergillus* species. There were no statistically significant differences in antifungal agent susceptibility between IB and IC lineages within *Aspergillus flavus*. However, within different lineages of *Fusarium falciforme*, FF6-7-8 was more susceptible to NAT than FF1, 2, 4, and 5. These statistically significant species differences in antifungal agent susceptibilities within *Aspergillus* demonstrate the importance of accurate identification of the potential fungal pathogen to the species level.

However, we did not find a correlation between *in vitro* sensitivity testing, antifungal used clinically, and FK outcome in these horses (Table 3). This may suggest that the clinical relevance of *in vitro* fungal testing is low and that additional methods are needed for better translate these results to clinical fungal keratitis. This subject is being currently investigated by our laboratories. Factors other than drug susceptibility may influence outcome in these clinical patients, such as variability of disease severity and host response to injury (e.g., host immune response and healing rates). Therefore, larger case numbers, MLST identification, and susceptibility testing are needed.

Further study of these equine FK isolates against other common antifungal agents is needed, including itraconazole, amphotericin B, clotrimazole, ketaconazole, and econazole. One study of *Aspergillus* from human FK demonstrated that *A*. *flavus* was susceptible to econazole, clotrimazole and ketoconazole while *A*. *fumigatus* was susceptible to amphotericin B, natamycin, voriconazole, and itraconazole.[47] In another study, amphotericin B and natamycin where shown to be effective against species of *Fusarium*, while species of *Aspergillus* were sensitive to amphotericin B and itraconazole.[6] Homa *et al*. [42] reported that terbinafine, natamycin, and amphotericin B followed by voriconazole were the most effective antifungal drugs for the majority of *Fusarium* isolates from human FK. As a whole, the results from these published studies support our data, but suggest that amphotericin B and possibly itraconazole are two antifungals that should be evaluated against isolates of *Aspergillus* and *Fusarium* from equine FK. O’Donnell *et al*. [11] showed that human FK isolates of the FSSC phylogeny complex (19 isolates representing 18 species) were insensitive to 10 antifungal agents tested *in vitro*. In contrast, we found that FSSC complex composed of *F*. *falciforme* was susceptible to natamycin and thiabendazole, but less susceptible to voriconazole and terbinafine. MIC values for *Aspergillus* spp. obtained in this equine FK study match those reported for human FK; as examples, for *A*. *flavus* 0.7 and 33.5 versus 1 and 32 μg/ml [50] for voriconazole and natamycin respectively; for *A*. *fumigatus* 0.4 and 4.4 versus 0.5 and 4 μg/ml [50] for voriconazole and natamycin respectively. In the case of *Fusarium* spp., there are both similarities and differences between MIC values in this equine FK study with those obtained from human FK studies in part due to the high variability among human FK studies.[51, 52]

Although fungal species and evolutionary lineage were not associated with clinical outcome in this study, associations regarding antifungal agent susceptibility demonstrated the importance of identifying the potential fungal pathogen to the species and lineage levels and not just to the generic level. These results also suggest that antifungal agent treatment of equine keratitis should be tailored to the infecting fungi and that accurate fungal species identification is critical to determine response to therapeutic agents and for developing effective treatment recommendations. Therefore, it is recommended to perform MLST typing routinely in FK to help choose appropriate antifungal therapy based on likely susceptibility and with a large sample size, ultimately, predict outcome.

## Supporting information

S1 Table Sequences of PCR primers used for amplification and sequencing of *Aspergillus* and *Fusarium* fungi and length of target regions. The primers used for Sanger sequencing are underlined.
(DOCX)

S2 Table Thermocycler conditions for all loci amplified in *Fusarium* and *Aspergillus*. (DOCX)
