# Supplementary material for: Multi-locus DNA sequence analysis, antifungal agent susceptibility, and fungal keratitis outcome in horses from Southeastern United States
Source: PLoS One. 2019 Mar 28;14(3):e0214214. doi: 10.1371/journal.pone.0214214 (PMC6438541; doi:10.1371/journal.pone.0214214)
Supplement: S1 Table — The primers used for Sanger sequencing are underlined. (DOCX) [file pone.0214214.s001.docx]

**Supplementary Table 1**. Sequences of PCR primers used for amplification and sequencing of *Aspergillus* and *Fusarium* fungi and length of target regions.

| Types | Loci | Sequence  5’-3’ | PCR & Sequencing Primer* | Length (bp) |
| --- | --- | --- | --- | --- |
| Both | ITS+LSU | ITS1: TCCGTAGGTGAACCTGCGG  LR3:  CCGTGTTTCAAGACGGG | ITS1 and LR3 | 1009-1153 |
| *Fusarium* | RPB1 | F7: CRACACAGAAGAGTTTGAAGG  R9: TCARGCCCATGCGAGAGTTGTC | F7 and R9 | 2100 |
|  | RPB2 | 5F2:  GGGGWGAYCAGAAGAAGGC  11aR: GCRTGGATCTTRTCRTCSACC | 5F2 and 11aR | 1742 |
|  | RPB2 | 7cF: ATGGGYAARCAAGCYATGGG  11aR: GCRTGGATCTTRTCRTCSACC | 7cF and 11aR | 860 |
| *Aspergillus* | aflM/aflN | aflM:  GCTTGGCTCTCTCCTTTGAA  aflN:  GCTGCTGAGGGAGTTGAAAC | aflM and aflN | 1200 |
|  | aflW/aflX | aflW:  GCACACGGTGTGGAAAGATA  aflX:  GACTAGTGCACGATGTGCAAC | aflW and aflX | 950 |
|  | trpC | trpC-F:  GACGGGAAATAGGCTTCC  trpC-R:  CGC CTT GGT GGG ATG GTG | trpC-F and trpC-R | 500 |
|  | XC4 (mfs) | XC4-F:  ATCGTGCAGACAGGAACAC  XC4-R:  GGTGCCTTGGCCTATGCGCT | XC4-F and XC4-R | 500 |
|  | amdS | amdS-F:  CCATCGGTATAGGAACTGA  amdS-R:  AGGGTGCCACGGTATGTC | amdS-F and amdS-R | 550 |
|  | MAT1-1 | M1F:  ATTGCCCATTTGGCCTTGAA  M1R:  TTGATGACCATGCCACCAGA | M1F and M1R | 400 |
|  | MAT1-2 | M2F:  GCATTCATCCTT TATCGTCAGC  M2R:  GCTTCTTTTCGGATGGCTTGCG | M2F and M2R | 270 |

*The primers used for Sanger sequencing are underlined.
