# Supplementary material for: Multi-locus DNA sequence analysis, antifungal agent susceptibility, and fungal keratitis outcome in horses from Southeastern United States
Source: PLoS One. 2019 Mar 28;14(3):e0214214. doi: 10.1371/journal.pone.0214214 (PMC6438541; doi:10.1371/journal.pone.0214214)
Supplement: S2 Table — (DOCX) [file pone.0214214.s002.docx]

**Supplementary Table 2**. Thermocycler conditions for all loci amplified in *Fusarium* and *Aspergillus.*

| Locus | Initial Denature | Denature | Anneal | Extension | # of Cycles | Final Extension | Hold |
| --- | --- | --- | --- | --- | --- | --- | --- |
| trpC  amdS | 95°C  2 mins | 95°C  1 min | 58°C  1 min | 72°C  1 min | 35 | NA | 4°C |
| mfs | 95°C  5 mins | 95°C  30 secs | 62°C  1 min | 72°C  2 mins | 40 | 72°C  5 mins | 4°C |
| aflM/aflN  aflW/aflX | 95°C  5 mins | 95°C  30 secs | 62°C  30 secs | 72°C  1 min | 40 | 72°C  5 mins | 4°C |
| M1F/M1R  M2F/M2R | 95°C  5 mins | 95°C  30 secs | 58°C  1 min | 72°C  45 secs | 40 | NA | 4°C |
| ITS1/LR3  RPB1 F7-R9  RPB2 5f2-11ar  RPB2 7cf-11ar | 95°C  90 secs | 95°C  30 secs | 55°C  90 secs | 72°C  2 mins | 40 | 72°C  5 mins | 4°C |
